# Microstructure and Mechanical Properties of TA2/Q235 Laser Weld Joint with Copper Interlayer

**DOI:** 10.3390/ma16103838

**Published:** 2023-05-19

**Authors:** Liang Zhang, Qi Wang, Xiaolei Guo, Pan Chen, Yinling Wang, Chen Wang, Zhanxue Wang, Zongling Wang

**Affiliations:** College of Energy Engineering, Huanghuai University, Zhumadian 463000, China; w2638577918@gmail.com (Q.W.); guoxiaolei@huanghuai.edu.cn (X.G.); chenpan@huanghuai.edu.cn (P.C.); 18845101997@163.com (Y.W.); 15190591016@163.com (C.W.); 18730681978@163.com (Z.W.); 18736770703@163.com (Z.W.)

**Keywords:** microstructure, mechanical properties, laser weld, dissimilar metal, copper interlayer

## Abstract

For the dissimilar metal welding needs of TA2 titanium and Q235 steel, preliminary trials were conducted using laser welding methods, and the results showed that the addition of a copper interlayer and the bias of the laser beam toward the Q235 side allowed for an effective connection. The welding temperature field was simulated using the finite element method, and the optimum offset distance of 0.3 mm was obtained. Under the optimized parameters, the joint had good metallurgical bonding. Further SEM analysis showed that the microstructure of the bonding area between the weld bead and Q235 was a typical fusion weld pattern, while that of the bonding area between the weld bead and TA2 was in brazing mode. The microhardness of the cross-section showed complex fluctuations; the microhardness of the weld bead center was higher than that of the base metal due to the formation of a mixture microstructure of copper and dendritic Fe phases. The copper layer not involved in the weld pool mixing had almost the lowest microhardness. The highest microhardness was found at the bonding site of TA2 and the weld bead, mainly due to the formation of an intermetallic layer with a thickness of about 100 μm. Further detailed analysis revealed that the compounds included Ti_2_Cu, TiCu and TiCu_2_, showing a typical peritectic morphology. The tensile strength of the joint was approximately 317.6 MPa, reaching 82.71% of that of the Q235 and 75.44% of the TA2 base metal, respectively. The fracture occurred in the unmixed copper layer.

## 1. Introduction

The density of titanium and its alloys is about 58% of that of steel. They also have high strength, good ductility and toughness, excellent corrosion resistance and thermal stability, which have been widely used in the key components of aerospace aircrafts, ships, vehicles and other means of conveyances. The substitution of titanium alloys for steel materials brings significant lightweight effects and performance enhancement [1,2,3]. However, the materials and manufacturing costs of titanium alloys are higher than those of steel, only a partial replacement can be implemented in the short term to achieve a balance between cost and performance. This inevitably leads to the need to connect titanium alloys to steel. Mechanical connection methods, such as gluing, riveting and bolted connections, always have problems regarding operating temperature limitations, weight increments and poor sealing. Generally, fusion welding is a good choice for joining titanium alloys and steel [4,5,6]. Among these methods, TIG and MIG welding are not normally used due to the poor connecting quality caused by property differences of titanium alloys and steel and the production of brittle intermetallics [7,8,9]. Researchers have attempted to adopt laser welding and electron beam welding to join titanium alloys and steel. Angshuman applied Ni powder as a filler material to weld commercial pure titanium and 304 stainless steel with 1 mm thickness. The results showed that the Ni powder could effectively retard the metallurgical reaction between Ti and Fe, and the formation of Ti-Fe intermetallic compounds had been inhibited. Meanwhile, the thermal expansivity of Ni was located between titanium and stainless steel, which weakened the welding stress and cracking tendency. Finally, the defect-free joint with an ultimate tensile strength of 375 MPa was obtained [10]. Gao used a 1 mm thick AZ31B magnesium alloy as the interlayer for the butt laser welding of Ti-6Al-4V and 304 stainless steel. Only the interlayer melted during the welding process due to its low melting point, and the joint was in brazing mode. The thin intermetallic layer appeared at the contact position between the base metal and weld on both sides. The tensile strength reached 221 MPa [11]. Zhang performed the laser welding on a 1 mm thick TC4 titanium alloy and 304 stainless steel using a 0.2 mm thick 45Ag-30Cu-25Zn interlayer metal. The welding position was located on the stainless steel, 1.6 mm away from the middle. Hence, the interlayer metal was located at the heat-affected zone and was melted during welding. Finally, the brazing welding was realized, an intermetallic layer of Fe_3_Zn_2_ was formed at the interface between the weld and steel, and the layer of Ti_2_Cu and TiCu phase was formed at the titanium side. The thickness of the layer on both sides was relatively thinner than 1μm; the joint tensile strength reached 284 MPa and broke at the interface between the steel and weld [12]. Wang attached the 1 mm thick Cu sheet to the joint of a TA15 titanium alloy and 304 stainless steel and used spot welding, then electron beam welding on the joint. The intermetallic layer was generated on both sides. The titanium side was composed of Ti-Cu and Ti-Fe-Cu compounds, while the stainless steel side was mainly a TiFe_2_ phase, and the joint strength reached 224 MPa [13]. Tomashchuk prepositioned 0.5 mm thick copper foil on the butt joint of a Ti-9Al-4V alloy and 316 L stainless steel and studied the influence of the electron beam welding position on the microstructure and mechanical properties of the joint. The results showed that when the electron beam deviated towards the titanium, a thicker TiFe_2_ compound layer was formed at the interface between the weld and the titanium alloy. With the offset towards the steel side, the thickness of the TiFe_2_ layer decreased, while the Ti-Cu-Fe compound layer appeared. When the electron beam was biased 0.25 mm to the steel side, the joint strength reached a maximum of 350 MPa [14]. Generally, the current studies mostly used heat sources with high energy density, such as lasers and electron beams, and used Cu, Ni and other metals as interlayers to restrain chemical interaction between Ti and Fe. Furthermore, a good welded joint could be acquired with the inhibition formation of Ti-Fe intermetallic compounds. Alternatively, low melting point metals were used as the interlayer to obtain brazed welding joints. However, it was difficult to avoid the formation of Ti-Fe, Ti-Cu and Ti-Fe-Cu intermetallic layers, and these brittle compound layers were often the weakest part of the joint. This study combined the advantages of fusion welding and brazing. Laser welding was performed to join TA2 pure titanium and Q235 steel with an interlayer of 0.5 mm thick Cu foil. Taking advantage of the different melting points of the three metals, the laser position was precisely controlled based on finite element (FE) simulation to ensure that the steel side realized the fusion welding mode without the intermetallic layer and the titanium side realized the brazing mode. Therefore, a good connection between TA2 and Q235 was achieved due to the observable inhibition of intermetallic compounds.

## 2. Materials and Methods

**Figure 1:**
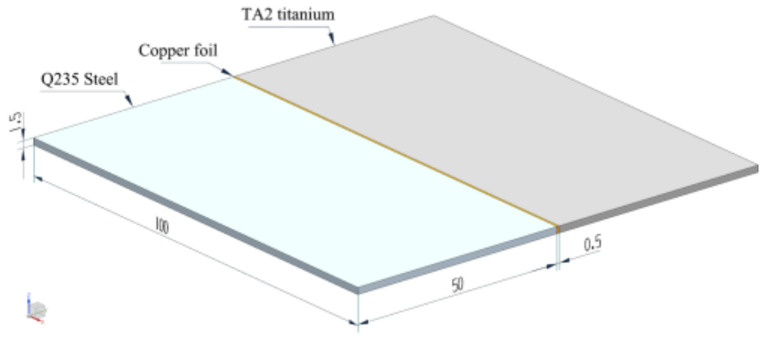
Dimensions of the welding specimen.

The base metals were the annealed TA2 titanium and Q235 steel plates with thickness of 1.5 mm. They were sheared into the size of 100 mm × 50 mm. Before welding, the joint was burnished using 600# abrasive paper. Then, it was cleaned with ethanol to remove the oxide skin and greasy dirt. T2 pure copper foil with thickness of 0.5 mm was selected as the interlayer metal. It was sheared into the size of 100 mm × 1.5 mm and then placed into the joint before welding. Figure 1 gives a schematic illustration of the joint. The chemical compositions of the base metals and interlayer are exhibited in Table 1. The mechanical properties of the base metals are shown in Table 2.

**Figure 2:**
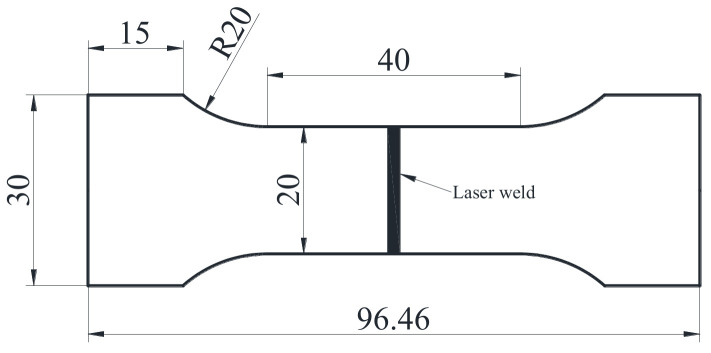
Dimensions of the tensile specimen.

A domestic automatic fiber laser welding machine with the model number of CW3000 was used for welding experiments. The machine was produced by Danyang Feichao Laser Technology Co., Ltd. in Zhenjiang, China. Its maximum power was 3000 W; the focal length was 300 mm. The laser spot diameter was adjustable between 0.2–2 mm. After welding directly or with the addition of the copper interlayer but no biasing, severe cracks appeared in the joints, so the weld bead was precisely extracted from the joint by means of wire electrical discharge machining. Then, the weld bead was carefully cut into small pieces using a small plate shearing machine. Then, these small pieces of weld bead were placed in the ball mill for grinding into powder suitable for XRD detection. The powder from weld bead was analyzed by X-ray diffraction (XRD) to determine the phases. For well-formed joints using optimized welding parameters, Vickers microhardness tests were carried out on cross-section of the joint using an HV-1000IS microhardness tester with load of 1.96 N and dwell time of 10 s. The microhardness tester was manufactured by Shanghai Jvjing Precision Instrument Manufacturing Co., Ltd. in Shanghai, China; the test point spacing was set as 0.1 mm. The microstructure was analyzed by the back-scatter electron imaging of TESCAN MIRA LMS scanning electron microscope (SEM, Brno, Czech Republic). Meanwhile, the micro-area composition was analyzed using Xplore type energy dispersive spectrometer (EDS, Oxford, UK) equipped in the SEM. Tensile specimens were prepared according to the GBT 2651-2008 standard, as shown in Figure 2. The tensile tests were conducted at a rate of 1 mm∙min^−1^ on an MIT-50 KN testing machine (made by Shenzhen Suns Technology Stock Co., Ltd. in Shenzhen, China), and every final test result was the average value of three tests.

## 3. Preliminary Welding Experiments

### 3.1. Welding Experiments without Interlayers

**Figure 3:**
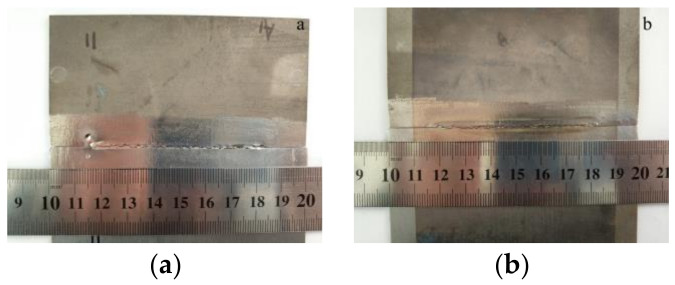
Macrographs of the weld joint without the interlayer: (**a**) top side; (**b**) back side.

Firstly, the laser welding of TA2 pure titanium and Q235 steel was performed without adding intermediate layer metal. The process parameters were 1200 W laser power, 1200 mm∙min^−1^ welding speed, 0 mm defocus distance and 15 L∙min^−1^ argon flow. The laser beam irradiated the butt joint without biasing. However, the longitudinal through-wall crack appeared immediately after the welding, as shown in Figure 3.

**Figure 4:**
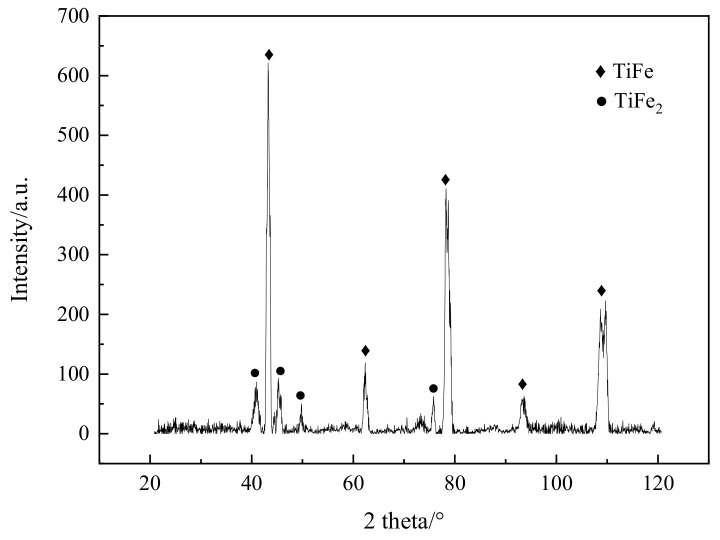
The XRD spectrum of weld bead without interlayer.

The XRD measurement of the weld bead was conducted for the phase determination, and the result is shown in Figure 4. Obviously, the weld bead was mainly composed of TiFe and TiFe_2_, which were brittle intermetallics and easily fractured due to the tensile stress during the cooling stage [15].

### 3.2. Welding Experiments with Interlayers

**Figure 5:**
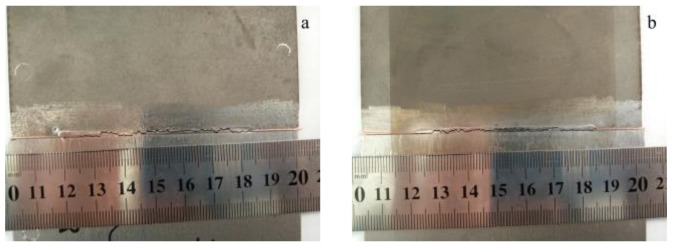
Macrographs of the weld joint with the interlayer but no biasing: (**a**) top side; (**b**) back side.

**Figure 6:**
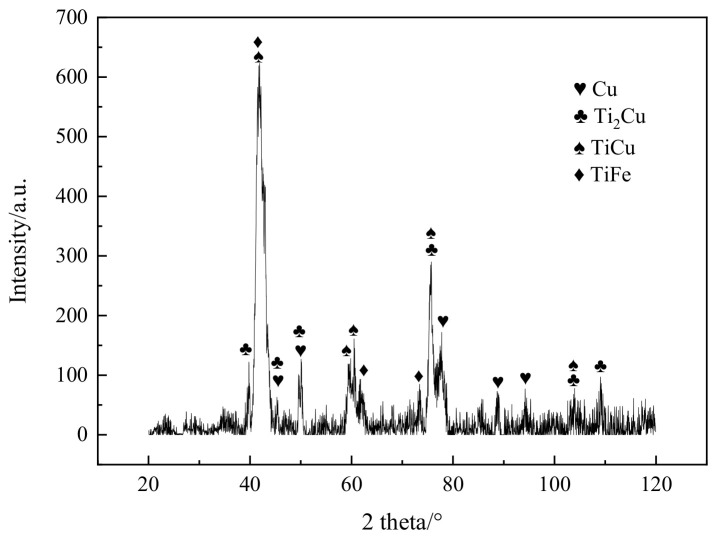
The XRD spectrum of weld bead with interlayer but no biasing.

Next, the laser welding was produced using the 0.5 mm thick copper interlayer, and the location of laser was the middle of the copper foil. Similarly, the weld bead was cracked integrally after welding, as shown in Figure 5. Its XRD spectrum is exhibited in Figure 6. A small amount of copper was detected due to the addition of copper interlayer. Furthermore, considerable phases of Ti_2_Cu, TiCu and TiFe were found. These compounds were so brittle and easily fractured because of tensile stress during the cooling stage [16].

**Figure 7:**
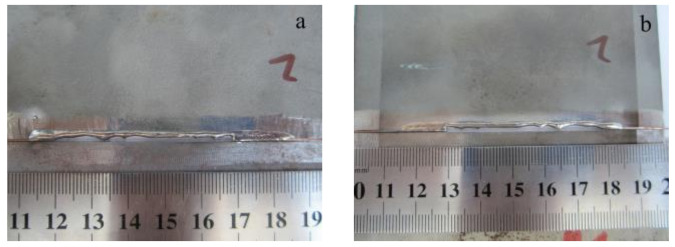
Macrographs of the weld joint with the interlayer and biasing to titanium: (**a**) top side; (**b**) back side.

Furthermore, laser welding with 0.5 mm thick copper interlayer and biasing of 0.2 mm to titanium was tried. The forming result is displayed in Figure 7. The specific heat capacities of titanium, steel and copper are similar, but their thermal conductivities are very different. The thermal conductivity of TA2 is about 14.63 W/m∙K, and those of Q235 and copper are about 83.74 W/m∙K and 397.51 W/m∙K, respectively. In other words, the welding heat diffused far away at the Q235 side, but it was suppressed at the TA2 side, which caused a localized melting of TA2, while the Q235 was unmelted [17,18,19].

## 4. Welding Experiments with Interlayer and Position Offset to Q235

### 4.1. FE Simulation of Temperature Field

The above attempts indicate that the bias of laser to Q235 may have a satisfying result. In order to determine the bias distance, FE simulations were performed. The geometric model consistent with the specimen was set up, and then the model was divided into the eight-node select-reduced element using the Hypermesh 2021 software. The elements adjacent to the weld were divided into about 0.05 mm. With the movement away from these regions, the mesh size gradually increased to several millimeters. Finally, the numbers of elements and nodes were 237,600 and 266,232, respectively. To simulate the laser welding process, classical three-dimensional Gaussian conical heat source whose accuracy had been widely confirmed was selected [20]. The convective heat loss and heat dissipation by radiation were considered in the simulation. The transfer coefficient of the convective heat loss was set as 20 W∙m^−2^∙K^−1^, while the surface emissivity was 0.8 [21]. The density, thermal conductivity and specific heat capacity of the materials were derived from the literature [22]. According to the actual welding parameters and thermal efficiency, the heat input was obtained as 54 J∙mm^−1^.

**Figure 8:**
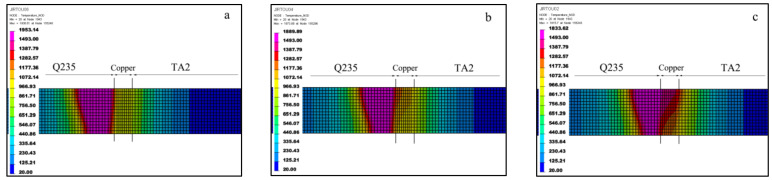
Simulation results of the welding temperature field with different offset: (**a**) 0.9 mm; (**b**) 0.6 mm; (**c**) 0.3 mm.

The positions of the laser beam were offset to the steel side as 0.9 mm, 0.6 mm and 0.3 mm, respectively. Results of temperature field simulations are exhibited in Figure 8.

Obviously, the weld pools were shown to be asymmetrical, mainly because of the differences in the thermophysical properties of the three metals. The pools were generally biased to the Q235 side because the thermal conductivity of Cu was much higher than that of the metals on either side, and the heat had been rapidly transferred. As shown in Figure 8a,b, a stable full melt-through weld pool was formed on the steel side, showing a typical laser welding pool morphology, similar to a wine glass. However, due to the larger offset, the Cu layers were not completely melted in the two conditions, that is, the temperature in the Cu regions did not completely reach the melting point. Moreover, in the case of the 0.9 mm offset specimen, the contact areas of the Q235 and Cu were barely melted. For 0.6 mm offset specimen, the melted region was increased, but there were still unmelted parts. On the other hand, when the offset was reduced to 0.3 mm, as shown in Figure 8c, the temperature on the steel side was above the melting point of 1493 °C, which means that the steel side was effectively melted. The temperature in the Cu layer was above its melting point of 1083 °C, which means that the Cu layer was also effectively melted. The titanium side was further from the heat source, and the temperature was lower than the melting point of 1660 °C, which means the titanium side remained completely solid phase during the welding process, while the copper interlayer was entirely liquid phase. Finally, the brazing mode at the titanium and Cu interface was obtained.

### 4.2. Macrographs of the Joint

**Figure 9:**
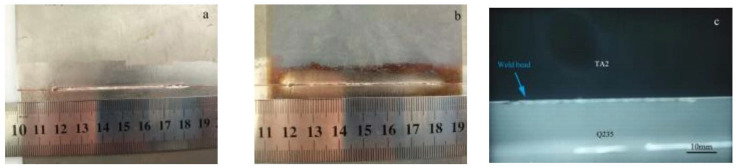
Macrographs of the weld joint with the interlayer and biasing to steel: (**a**) top side; (**b**) back side; (**c**) X-ray detection.

Based on the simulation results, the welding tests were performed using the strategy of the 0.3 mm offset to the steel side. The weld formation is shown in Figure 9. A good metallurgical combination was achieved. The weld bead appeared to be well-formed, with a uniform width and no surface defects such as cracks and unevenness. The top side of the weld was approximately 1.5 mm wide and slightly wider than the back side of 1 mm. The X-ray detection result is presented in Figure 9c. There were very few tiny pores in the weld, which could be considered to have little effect on the mechanical properties.

### 4.3. The Microhardness Distribution of the Joint

**Figure 10:**
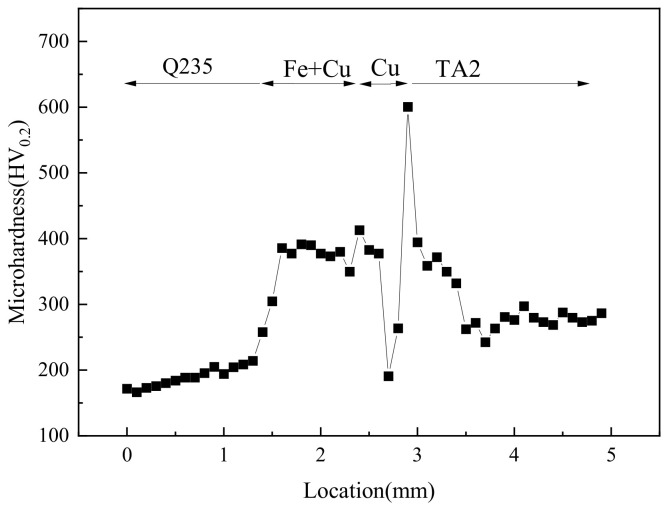
The microhardness distribution.

The cross-sectional microhardness distribution of the joint is shown in Figure 10. The microhardness shows a complex fluctuation. The microhardness of the Q235 was basically the lowest, averaging about 171.71 HV, while the TA2 had a higher hardness that reached approximately 278.52 HV. In the transition zone of the Q235 to weld bead, the microhardness gradually increased. The microhardness within the weld bead was essentially the same as approximately 381.63 HV. However, the Cu layer that was not involved in the weld pool mixing had a lower microhardness of about 190.72 HV. At the bonding site of the TA2 and Cu, the microhardness rose significantly to the maximum value of 600.49 HV. With the transition to the TA2 base metal, the microhardness gradually decreased to the level of the TA2.

### 4.4. Microstructure

**Figure 11:**
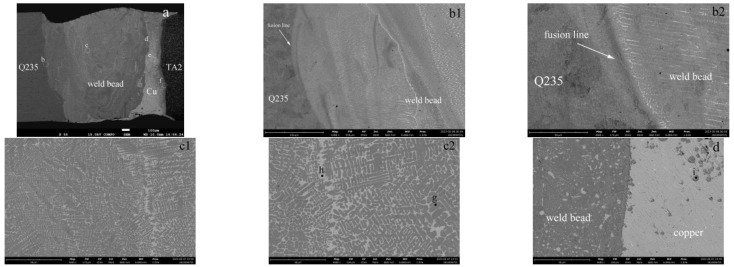

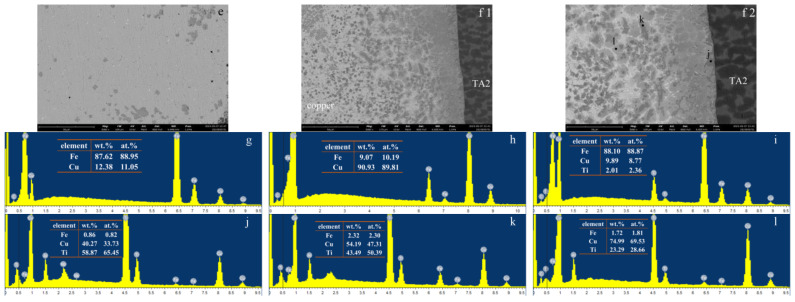
SEM images and EDS results of the welded joint: (**a**) entire joint; (**b1**) bonding area between the Q235 and weld bead with lower magnification; (**b2**) bonding area between the Q235 and weld bead with higher magnification; (**c1**) weld center with lower magnification; (**c2**) weld center with higher magnification; (**d**) bonding area between the weld bead and copper; (**e**) copper center; (**f1**) bonding area between the weld bead and TA2 with lower magnification; (**f2**) bonding area between the weld bead and TA2 with higher magnification; (**g**) Fe energy spectrum at the weld center; (**h**) Cu energy spectrum at the weld center; (**i**) Fe energy spectrum at the copper; (**j**) Ti_2_Cu energy spectrum; (**k**) TiCu energy spectrum; (**l**) TiCu_2_ energy spectrum.

As shown in Figure 11a, the weld bead was mainly created in the combination of the Q235 and Cu layer, which means the composition here was a mixture of Q235 and Cu due to the vigorous stirring during welding. The Q235 base metal achieved a good metallurgical bond with the copper interlayer, showing a typical fusion weld pattern. Based on Figure 11a, the area proportion of molten Q235 and Cu in the weld bead was further precisely calculated. Considering the density of Q235 and Cu, the mass ratio of Fe and Cu in the weld bead could be derived as approximately 2.6:1, which means the percentage of Fe was about 72.7%. Just as we expected, an unmixed copper layer was present between the weld bead and TA2. The different positions in the joint were further analyzed, and they were labeled as b, c, d, e, f, corresponding to bonding area between the Q235 and weld bead, weld center, bonding area between the weld bead and copper, copper center and bonding area between the weld bead and TA2, respectively.

Figure 11(b1) shows the bonding area of the Q235 and weld bead. A clear fusion line can be found separating the Q235 from the weld bead. The further magnified observation of this region is shown in Figure 11(b2). The grains in the weld bead grew attached to the Q235 boundary, showing a typical epitaxial solidification. These grains had obvious directionality towards the weld bead and exhibited a typical columnar dendrite morphology. The morphology of the weld center is shown in Figure 11(c1), consisting of a large number of dendritic crystals and a few intergranular phases. The magnification of this region is shown in Figure 11(c2). The dendritic crystals were dark gray, and the intergranular phases were light gray; they were labeled as g and h, respectively. The EDS results showed that they were Fe phases and Cu phases, respectively, as shown in Figure 11g,h. The bonding area of the weld bead and copper layer is shown in Figure 11d. There was an obvious boundary between them, and the irregularly shaped dark gray phases were sporadically distributed in the copper layer, as shown in position i. Its EDS result is shown in Figure 11i; the phases were mainly Fe ones. In the copper layer center, as shown in Figure 11e, a small number of Fe phases dispersed on the Cu matrix. Figure 11(f1) shows the bonding area of the copper layer and TA2. The interface between them was flat, and a layer of intermetallic compounds was generated adjacent to TA2, reaching a thickness of around 100 μm. When moving away from TA2, the number of compounds gradually decreases and soon transitioned to the copper layer. Further magnified observation of the compound layer revealed three main forms of compounds, as shown in Figure 11(f2). The dark gray compounds labeled as j appeared adjacent to TA2, whose dimensions were approximately 10 μm in length and 5 μm in width. The EDS result in Figure 11j showed that its composition was close to Ti_2_Cu. The slightly distant light gray compounds, marked as k, had tdendritic morphology up to 100 μm in length, and their composition was close to TiCu, as shown in Figure 11k. Another off-white phase was wrapped around TiCu phases, which showed a typical peritectic or peritectoid pattern. EDS results indicated that the compositions were close to TiCu_2_, as shown in Figure 11l.

**Figure 12:**
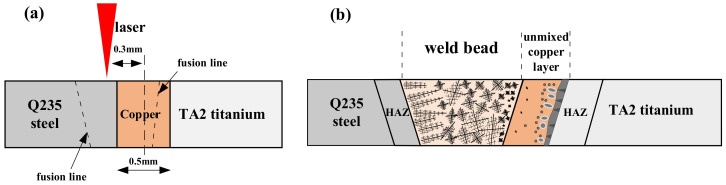
Schematic illustration of the joint forming: (**a**) welding process; (**b**) microstructure.

Based on the welding process analysis, the microstructure evolution could be obtained, as shown in Figure 12a,b. Under laser irradiation, the weld pool was established between Q235 and copper layer. Fusion lines were located in Q235 and the copper layer, respectively. The main composition of the weld bead was 72.7% Fe and 27.3% Cu in mass ratio. Combined with the Cu-Fe phase diagram, the microstructure evolution could be acquired [23]. Above the liquidus temperature, Cu and Fe co-existed as homogeneously mixed liquid phases. As the temperature dropped below the liquidus temperature, the weld bead accessed the liquid miscibility gap, which meant the mixed liquid separated into two liquid phases: an Fe-rich liquid phase and a Cu-rich liquid phase [24]. The weld pool was solidified very quickly, so this metastable microstructure was preserved. As Fe had a higher melting point than Cu, when the liquid was cooled to the miscibility gap, Fe had greater undercooling and was more likely to separate out of the mixed liquid. Continuing to cool to below the miscibility gap, γ-Fe was precipitated and attached to Q235 boundary, and dendrites grew in a columnar pattern in the weld pool due to the excellent heat dissipation paths provided by Q235 border, as shown in Figure 12b. On the other hand, the dendritic γ-Fe could also be precipitated directly from the liquid. Owing to the extremely fast cooling of the weld pool, the solid-liquid interface fronts had a larger composition undercooling, which facilitated the growth of dendritic γ-Fe so that lots of dendritic γ-Fe would be retained in the weld bead center.

Cooling continued to the peritectic temperature of about 1096 °C, which was still above the melting point of Cu. At this point, the Cu was still all present in the liquid phase between the dendritic γ-Fe. The liquid Cu and the dendritic γ-Fe would underwent peritectic reaction to produce solid phase Cu around the γ-Fe. However, under rapid cooling conditions, the peritectic reaction did not proceed sufficiently, and the solid Cu might have precipitated directly from the liquid with a higher solution of Fe than that in the equilibrium. The γ-Fe continued to cool rapidly, and a martensitic transformation probably occurred. The dendritic Fe phase may have been present in the form of martensite at room temperature, which ensured higher hardness in the weld bead than the Cu and Q235 base metal. So, at room temperature, the weld bead was composed of a large amount of dendritic Fe and Cu distributed between them, as shown in Figure 11(c1).

During welding, a small portion of the copper layer melted but did not participate in the vigorous stirring of the weld pool, which was called unmixed copper layer, as shown in Figure 12b. The bonding area of the weld bead and this layer was just the boundary of the weld pool, where the flow rate was low [25]. So, a clear boundary could be formed here, as shown in Figure 11d. On the other hand, due to the higher melting point of TA2 and the fact that it was farther from the laser heat source, the TA2 base metal was in a solid state during welding. So, a brazed joint was produced between the unmixed copper layer and TA2, as shown in Figure 12b. The Ti-Cu phase diagram may be useful to understand this morphology. As the distance from TA2 increased, the Cu content gradually increased, and intermetallic compounds such as Ti_2_Cu, TiCu and TiCu_2_ were generated in turn. The liquid phase could react with Ti_2_Cu to generate TiCu, and the liquid phase could also react with TiCu to generate TiCu_2_, so the intermetallic compounds layer showed TiCu_2_ wrapped in TiCu and TiCu wrapped in Ti_2_Cu. Precisely, the presence of this intermetallic compounds layer led to a significantly elevated hardness of about 600.49 HV.

### 4.5. Mechanical Properties

**Figure 13:**
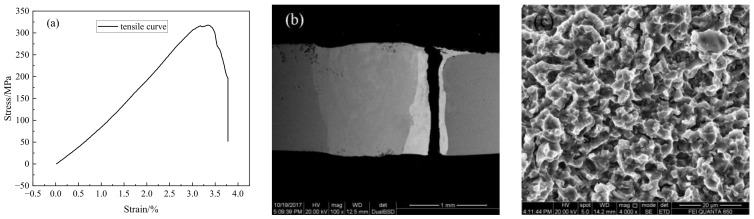
The tensile curve and fracture morphology of the joint: (**a**) tensile curve; (**b**) fracture position; (**c**) fracture surface.

Tensile tests for joints with optimized welding parameters were conducted. The representative tensile curve is illustrated in Figure 13a. The tensile strength of the joint was approximately 317.6 MPa, reaching 82.71% of that of the Q235 and 75.44% of the TA2 base metal, respectively. The weld joint coefficient was about 0.83. However, the plastic deformation stage in the curve was very short, which means the plasticity was not so good. As the unmixed copper layer was almost the weakest region in the joint, cracks emerged and propagated here until fracture, as shown in Figure 13b. This suggests that it may be possible to improve the strength by adjusting the thickness or composition of the copper layer. Unfortunately, very few plastic deformations can be observed near the fracture. The fracture appearance observed by SEM is shown in Figure 13c. There were almost no visible defects, such as hot cracking and pores. Lots of very small and shallow equiaxial dimples were observed, which indicated that the mechanism of fracture was mainly microvoid coalescence fracture. Meanwhile, these shallow dimples also indicated that the plasticity of the joint was not very good. The main reason may be the small thickness of the unmixed copper layer and the fact that its plastic reserve was limited during the tensile test. In further studies, it could be possible to enhance the plasticity by increasing the thickness of the copper layer.

## 5. Conclusions

We aimed to solve the problems of the high production costs and poor mechanical properties of titanium/steel dissimilar metal fusion welding. The optimized welding parameters and laser position were obtained through welding experiments and FE simulations. Finally, a mixture microstructure of fusion and brazing welding was achieved. The joint showed excellent mechanical properties. By analyzing the microstructure and mechanical properties of the joint, the metallurgical bonding mechanism was obtained. The following conclusions were obtained.

(1) Laser welding of TA2/Q235 dissimilar metals directly or with the addition of the copper interlayer but no biasing both resulted in penetration cracks after welding, which was mainly related to intermetallic compounds in the weld bead.

(2) Under the optimized welding parameters, the joints were well-formed, and a good metallurgical combination was achieved. The joining between Q235 and the copper layer was a typical fusion weld pattern. Meanwhile, the bonding of the copper layer and TA2 was a typical brazing pattern.

(3) The unmixed copper layer had almost the lowest microhardness in the joint, and cracks emerged and propagated here under tensile tests. The joint had an excellent tensile strength, with the joint coefficient of about 0.83.

## Figures and Tables

**Table 1:** Chemical compositions of the experimental materials (wt.%).

	Fe	C	Mn	Cu	Si	Ti	P	S	O
Q235	99.12	0.16	0.56	0.02	0.08	-	0.016	0.009	0.03
TA2	0.2	0.07	-	-	-	99.84	-	-	0.015
Cu	0.008	-	-	99.96	0.004	-	0.007	0.009	0.01

**Table 2:** Mechanical properties of the base metals.

	Yield Strength/MPa	Tensile Strength/MPa	Percentage Elongation/%
Q235	249	384	31
TA2	341	421	35

## Data Availability

The data are available from the corresponding author upon reasonable request.
